# *In Crystallo* Photochemistry: Reimagining Synthetic Tractability with Transparent Single-Crystalline Flasks

**DOI:** 10.1021/acscentsci.5c00549

**Published:** 2025-05-19

**Authors:** Aishanee Sur, David C. Powers

**Affiliations:** Department of Chemistry, Texas A&M University, College Station, Texas 77843, United States

## Abstract

Expanding the boundaries of synthetic tractability — of what molecules can be synthesized and isolated — is an eternal challenge for synthetic chemists. The development of new synthetic methods and strategies enables the properties and potential functions of novel molecular targets to be experimentally evaluated. In the context of catalysis, predictable synthetic strategies are often available to access kinetically persistent intermediates such as catalyst resting states. In contrast, synthesis and characterization of the reactive intermediates are often not possible due to the fleeting lifetimes of these species. *In crystallo* photochemistry combines single-crystal matrix isolation with cryogenic photochemistry to enable reactive intermediates to be synthesized under conditions in which they are persistent and can be (crystallographically) characterized. This Outlook highlights key achievements of *in crystallo* photochemistry as well as discusses opportunities and challenges that confront realization of the potential of *in crystallo* synthesis to redefine the boundaries of synthetic tractability.

## Graphical abstract

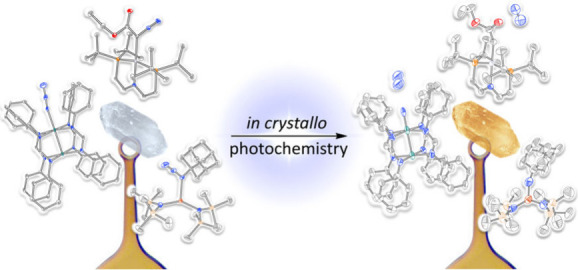

## 1 Introduction

To evaluate the properties and potential applications of hypothetical small molecules, such as pharmaceutical candidates, target structures must be accessible via predictable synthetic sequences and need to be sufficiently stable to allow isolation and characterization (i.e., synthesizable or synthetically tractable).^1–3^ As such, the continued development of novel synthetic methods to expand the chemical landscape by pushing back the boundaries of current synthetic tractability is a persistent forefront challenge. In the context of catalysis, myriad tools are available to access diverse precatalyst structures and catalyst resting states, which represent kinetically persistent, thermodynamic minima in catalytic cycles. Despite the critical role reactive intermediates have in determining reaction selectivity and catalyst efficiency, tools to experimentally access post-turnover-limiting step intermediates and other transient species relevant to catalytic cycles are much more limited. This challenge is in large part due to the lack of synthetic strategies to generate highly reactive species under conditions in which they are sufficiently persistent to be isolated and structurally characterized.

The last 10 years have witnessed the emergence of *in crystallo* photochemistry as a tool for molecular synthesis.^4–7^ *In crystallo* photochemistry combines cryogenic synthetic photochemistry with solid-state molecular synthesis to enable the synthesis and characterization of reactive intermediates and other exotic molecular structures.

### Synthetic Photochemistry

Reactive intermediates are inherently challenging to observe during thermally promoted reactions, because the same thermal energy used to generate them contributes to rapid decomposition processes. In concept, synthetic photochemistry, which proceeds on excited state potential energy surfaces and thus avoids thermal barriers on the corresponding ground state surface, can be carried out at sufficiently low temperature to enable the trapping of photogenerated reactive species. This strategy has enjoyed enormous historical success: Cryogenic matrix isolation studies, in which photoreactions are carried out at very low temperature in inert reaction matrices (e.g., frozen Ar, Kr, or Ne) have enabled synthesis and observation of extremely reactive species.^8,9^ For example, imidogen (i.e., NH), which is the simplest inorganic nitrene and transient under ambient conditions, can be observed by IR spectroscopy following photolysis of HN_3_ at 12 K in an Ar matrix.^10^ In matrix isolation studies, reactive species are persistent due to the inert reaction matrix and low temperature synthesis and thus can be spectroscopically observed. At the same time, the need for cryogenic matrix isolation limits the potential characterization tools that can be applied: Typically, only vibration spectroscopy and EPR measurements are available.^11^

### Solid-State Molecular Synthesis

In solution-phase reactions, the solvent provides a medium for reactants to encounter one another and can impact the course of a reaction by solvating reagents, products, and transition states. Those same solvent interactions present challenges for the synthesis of reactive species: Solvent coordination to metal centers prevents access to unsaturated metal ions and competes for coordination sites with weakly donating ligands; solvent functionalization reactions can limit the lifetime of reactive species by providing facile reaction pathways.^12,13^ Crystallization provides a unique chemical environment that 1) excludes solvent and other potentially reactive small molecules, and 2) reduces the degrees of freedom of lattice-confined species.^14,15^ As such, species that are transient in the solution phase can be generated and isolated in the crystalline phase.

Weller et al. have demonstrated this concept beautifully in the context of σ-alkane complexes, which are generated by *in crystallo* hydrogenation of metal olefin complexes.^16–19^ Because the critical hydrogenation reaction is carried out within a single crystal, the resulting complexes can be characterized by X-ray diffraction methods. Because molecular crystals are usually nonporous (i.e., van der Waals crystals), a limited variety of reagents diffuse into the crystalline sample, thus a limited set of reactions can be routinely employed. The small size of H_2_ enables *in crystallo* hydrogenation; challenges remain to accessing non-hydrogenation based mechanisms in solid-state molecular synthesis.^20^ Pioneering work by Fujita et al. demonstrated diffusion of large substrates and reagents within coordination networks and other porous materials,^21–24^ but application of extended networks as a general platform for molecular synthesis remains a challenge.

Merging cryogenic photochemistry with single crystal reaction environments provides a unique opportunity for molecular synthesis of reactive species.

Photochemical reactions within molecular single crystals (i.e., crystalline flasks) offer routes to synthesize new, potentially reactive molecular fragments within a confined environment. Because crystallography experiments are usually carried out at a low temperature (i.e., 100 K or lower), reactive species can persist.^4–7^ Early examples of *in crystallo* photochemistry demonstrated photopromoted linkage isomerism reactions within single crystals. For example, Raithby et al. demonstrated photoisomerism of Ni­(II) (η^1^-NO_2_) nitro ligands to metastable (η^1^-ONO) nitrito species (Figure 1a).^25^ Subsequently, *in crystallo* synthesis was extended to irreversible photoreactions, first using crystalline samples stabilized by ion pairing — e.g., Ohashi et al. demonstrated the preparation of organic nitrenes by photoactivation of carboxylate-tagged aryl azides (Figure 1b)^26^ — and more recently in the context of van der Waal’s crystals — e.g., Powers et al. described the preparation of reactive Ru_2_ nitride **6** (Figure 1c).^27^ This Outlook summarizes recent successes of *in crystallo* photochemistry and discusses forefront opportunities (and challenges) that have the potential to reimagine the synthetic tractability of reactive molecular targets.

**Figure 1:**
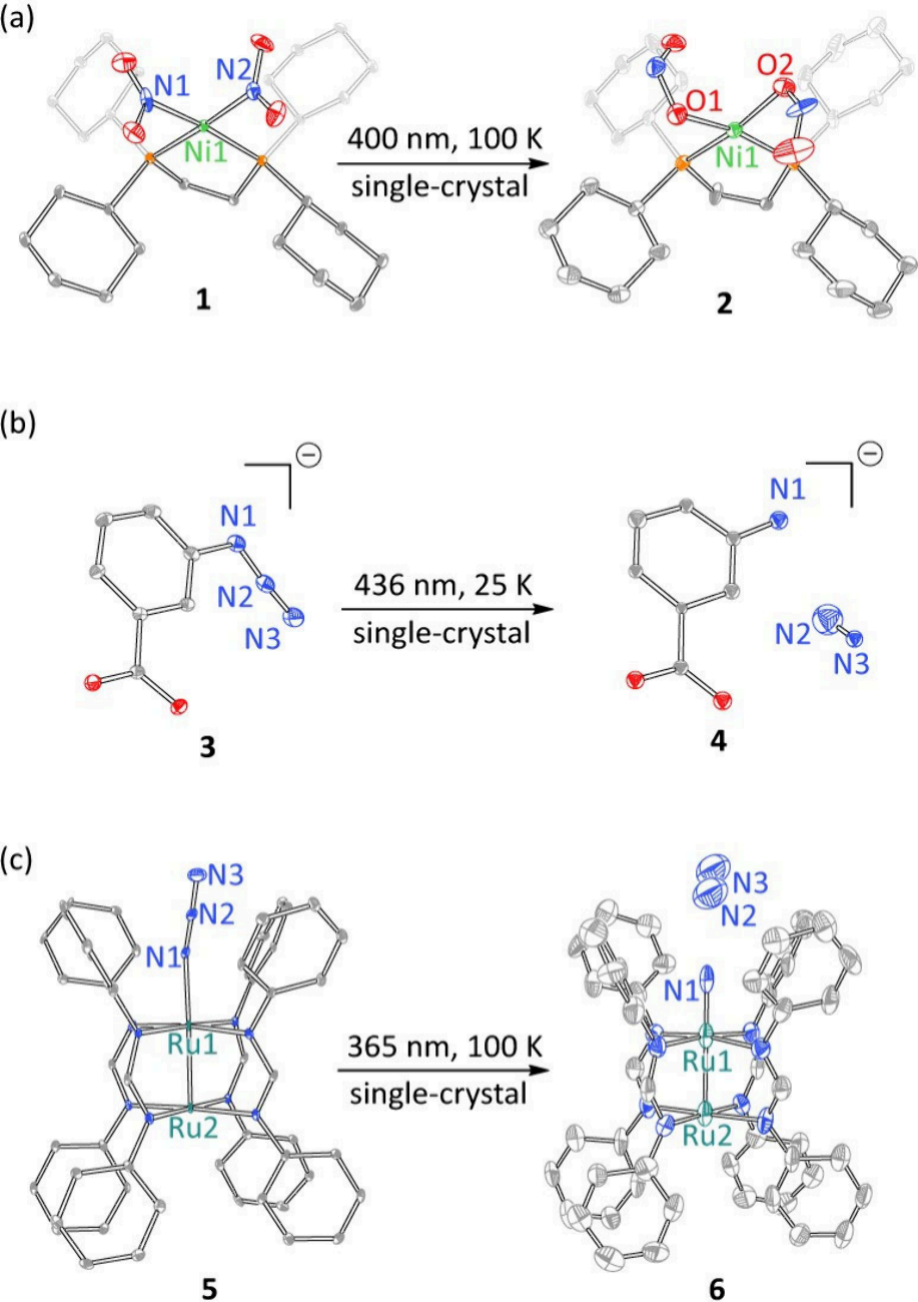
Early examples of *in crystallo* photochemistry (H atoms omitted for clarity): (a) Photodriven *N*-to-*O* linkage isomerism of Ni–NO_2_ complex **1**, (b) arylnitrene photogeneration within an ionic crystal lattice ((PhCH_2_)_2_NH_2_^+^ cations omitted for clarity), and (c) Ru_2_ nitride synthesis within a van der Waals crystal (Cl atoms omitted for clarity).

## 2 *In Crystallo* Synthetic Photochemistry

### 2.1 Experimental Considerations

*In crystallo* photochemical experiments begin with preparation of an appropriate single-crystalline sample of the photoactive molecule of interest. Ideal single crystals are typically 10−50 μm, with thin crystals and larger surface areas preferred to optimize light penetration while maintaining diffraction quality, as crystallinity often degrades during *in crystallo* photoreactions. Single crystal samples are mounted on standard goniometers and cooled to an appropriate temperature; most of the results highlighted here are collected between 80−100 K. The crystalline sample is irradiated, for example, by LED source of appropriate wavelength, and reaction progress is monitored by periodic collection of diffraction data. Structural refinement is performed by treating the diffraction data as the superposition of the dark and light states. Sufficient photoconversion is crucial for reliably identifying photoproducts, in particular, when light atoms such as C, N, or O are primary sites of interest: For example, at 10% photoconversion of a metal azide to the corresponding nitride, the photogenerated nitride ligand would only contribute 0.7 electron, which is close to the detection threshold in most crystallographic experiments. Further, potential disorder of small molecules (e.g., N_2_) generated during the *in crystallo* photoreaction can lower the R2 factor of the resulting refinement.^28^

### 2.2 Capturing Post-Turnover-Limiting Step Intermediates

Solution-phase synthetic chemistry provides a rich toolbox of methods to prepare and isolate kinetically persistent intermediates in catalytic cycles such as the catalyst resting state. Despite the critical role that post-turnover-limiting step intermediates have on reaction selectivity and the intimate role of these species in bond-making and -breaking steps of catalysis, these fleeting species are much less commonly observed.^29^ In the absence of synthetic access to reactive intermediates in catalysis, computational evaluation of relevant potential energy surfaces is benchmarked against metrical parameters of precatalysts and resting states that may not be appropriate proxies for more reactive catalytic intermediates.

*In crystallo* photochemistry provides a platform to synthesize and characterize post-turnover-limiting step intermediates. For example, kinetics experiments implicate Rh nitrenes in C–H amination catalysis, but these species are unobservable by *in operando* methods because they are formed after the turnover-limiting step (i.e., vanishingly low steady state concentration during catalysis).^30^ In 2019, Powers et al. demonstrated that reactive Rh_2_ nitrenes could be accessed by photoextrusion of N_2_ from Rh_2_ complexes of organic azides (Figure 2a).^31,32^ These studies provided the first demonstration that *in crystallo* synthesis can enable the characterization of transient catalytic intermediates and provided the first experimental evidence that Rh_2_ nitrenes display triplet ground states. In 2023, Chang et al. beautifully extended these studies to acyl nitrene fragments, which were generated by photoextrusion of CO_2_ from Rh dioxazolone precursors.^33^ Together, these studies of Rh nitrene structure provide experimental benchmarks for computational investigations, demonstrate the synthetic tractability of post-turnover-limiting step intermediates, and highlight the promise of *in crystallo* photochemistry to impact catalysis science.

**Figure 2:**
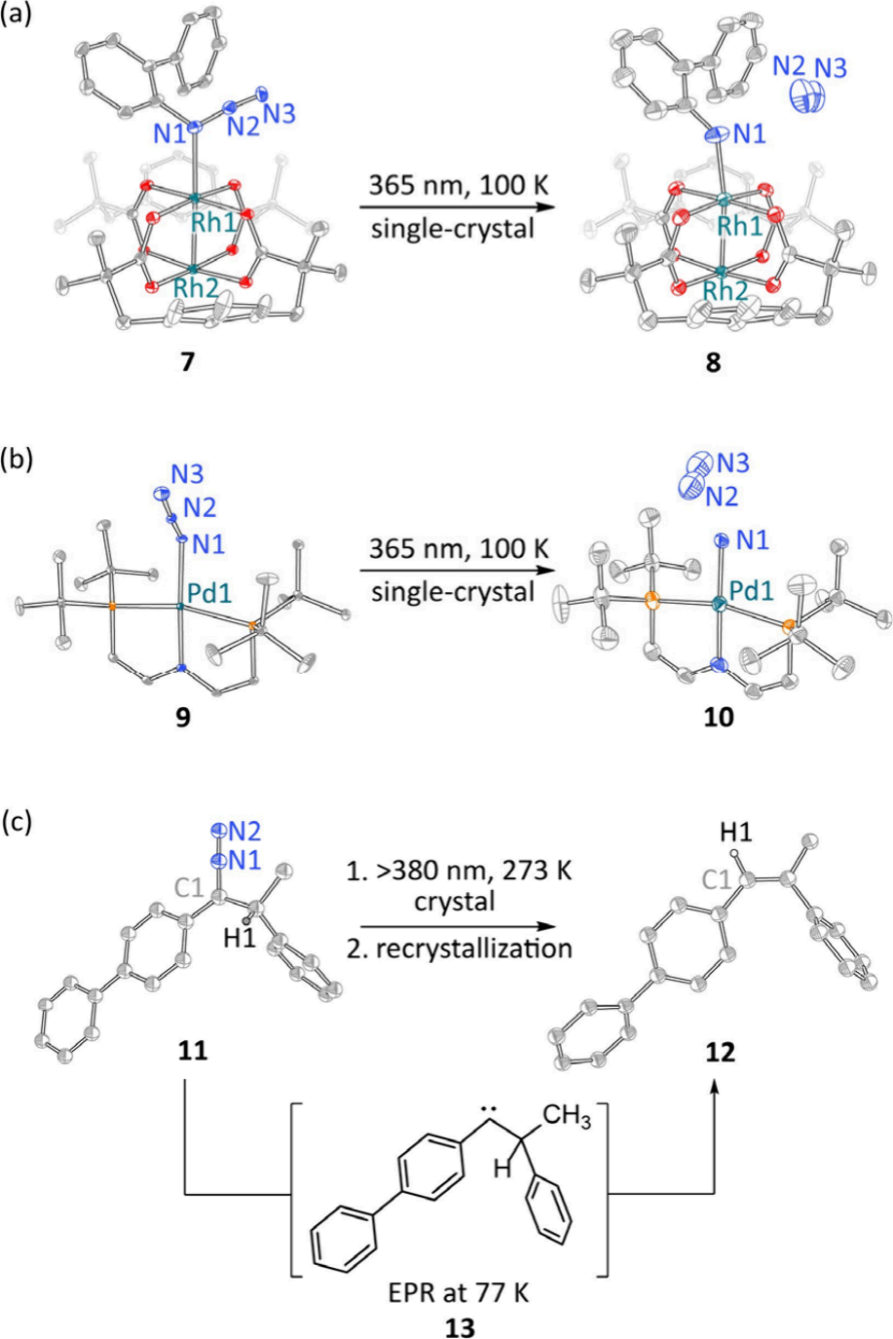
*In crystallo* photochemistry for (a) observation of post-turnover-limiting step intermediates in catalysis, (b) characterization of exotic electronic structures, such as triplet metallonitrene **10**, and (c) lattice-induced structural distortions. H atoms omitted for clarity.

### 2.3 Isolating Exotic Electronic Structures

High-spin, open-shell intermediates are implicated in many challenging bond-forming reactions in Biology.^34^ The electronic structures of these species give rise to the observed reactivity: High-spin configurations result from population of antibonding molecular orbitals. The importance of open-shell intermediates in Biology, coupled with the emergence of small-molecule catalysis that leverages high-spin intermediates for synthetic chemistry, motivates ongoing efforts to synthesize, isolate, and characterize high-spin metal–ligand (M–L) fragments.^35–37^ Classical strategies based on steric protection of the reactive site have enabled isolation of persistent complexes, such as Cu nitrenes^38^ and persistent triplet bismuthinidenes,^39^ but introduction of steric protection inherently attenuates the reactivity of the obtained fragments.

Application of *in crystallo* methods provides the opportunity to isolate exotic electronic structures without synthetic modification.

Holthausen, Powers, Schnegg, and Schneider et al. utilized *in crystallo* chemistry to isolate a family of metallopnictinidenes that feature electronically unsaturated, triplet atomic N, P, and As ligands.^40–42^ For example, N_2_ photoextrusion from Pd azide **9** affords triplet metallonitrene **10** (Figure 2b), in which two electron holes are localized on the atomic nitrogen ligand.^40^ Betley similarly applied *in crystallo* photochemistry to the synthesis of reactive open-shell Fe nitrides.^43^ Similarly, a small but growing family of triplet carbene complexes have been accessed by *in crystallo* photochemistry.^44–46^ While in most cases, persistent carbene complexes display singlet ground states,^47^ the combination of crystalline matrix isolation and cryogenic photolysis has enabled characterization of the correspondingly more reactive triplet carbene analogues. Together, these studies demonstrate the potential for *in crystallo* photochemistry to redefine the diversity of chemical and electronic structures amenable to isolation and characterization.

### 2.4 Trapping Distorted Geometries

*In crystallo* reactions are carried out within crystal lattices that are assembled from photoprecursors, not the photoproducts, and thus the lattice that *in crystallo* reactions are carried out in is not optimized for the analyte of interest. Confinement within a crystal lattice restricts molecular degrees of freedom and can limit the ability of a photogenerated molecule to relax to ground state geometries that would be accessed in the solution or gas phase.^48^ The potential strain implied by lattice confinement represents an opportunity to access distorted geometries that may be inaccessible in solution. García-Garibay et al. highlighted this possibility during *in crystallo* studies of carbene rearrangements (Figure 2c).^49,50^ *In crystallo* N_2_ elimination from **11** afforded 1,2-diphenylethylidene (**13**), which was characterized by EPR spectroscopy (but not crystallographically). In the solid state, rearrangement of **13** to **12** is much slower than expected based on comparison to solution-phase measurements. The depressed reactivity was attributed to crystal-induced distortion of compound **13**: Diazo precursor **11** crystallizes with an R–C­(N_2_)–R angle of 126°; in the gas phase, the singlet and triplet configurations of carbene **13** are calculated to display angles of 117° and 137°, respectively. Confinement-induced population of the less-reactive triplet was suggested to account for the observed rate inhibition. Similar lattice-induced geometrical distortions have been noted by Nocera during *in crystallo* studies of halogen-elimination photochemistry from Rh_2_ complexes,^51^ by Powers in the study of adamantyl nitrene complexes of Rh_2_,^31^ and by Powers and Severin during studies of Cu alkenylidenes.^44^

## 3 Opportunities and Challenges

The emergence and early successes of *in crystallo* photochemistry have already impacted synthetic chemistry, mechanism elucidation, and catalysis science. Here, we describe areas that we perceive *in crystallo* photochemistry has enormous potential to further impact molecular synthesis and highlight challenges that must be overcome to realize that potential.

### 3.1 Recording Molecular Movies

A dream of mechanistically inclined scientists is to experimentally plot the course of chemical transformations with atomic resolution. If multistep-reaction sequences—via sequential photochemical reactions or via temperature-programmed reactions of photogenerated species—could be achieved, *in crystallo* synthesis would provide a series of crystallographic still frames along a reaction coordinate that begin to define a molecular movie.

Tantalizing progress toward this goal has begun to emerge. Two recent studies provide structural characterization of species along the reaction coordinate of C–H activation. During studies of halogen elimination from Fe­(III) chloride **14**, Nocera et al. observed the *in crystallo* synthesis of Fe­(II), HCl, and a ligand-borne *C*-centered radical (Figure 3a).^52^ These products presumably arise from a two-step *in crystallo* reaction sequence of 1) Fe–Cl photocleavage to generate Fe­(II) and chlorine radical and 2) H-atom abstraction. Münz et al. also described *in crystallo* C–H activation (Figure 3b).^46^ In this example, N_2_ extrusion from a Pb diazo complex **16** afforded an observable triplet carbene intermediate. Photolysis of a crystal of **16** that contained a toluene molecule in the unit cell afforded C–H activation product **17**.

**Figure 3:**
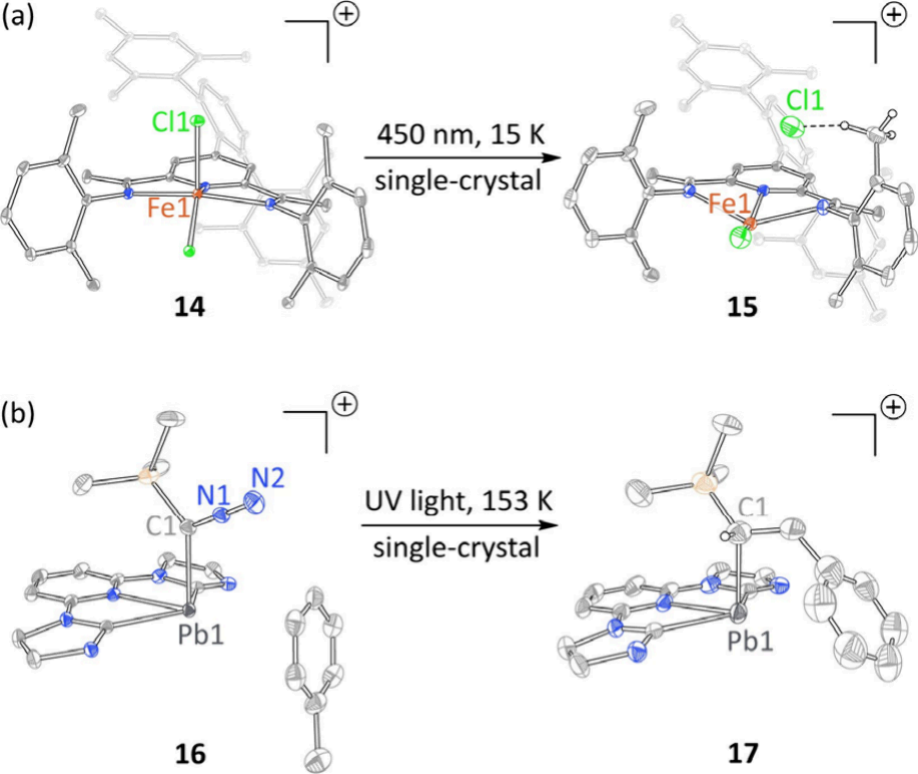
*In crystallo* C–H activation via (a) photogenerated chlorine radicals (H atoms and PF_6_^–^ counteranion omitted for clarity) and (b) photogenerated triplet carbene complexes of Pb (H atoms and [BAr^F^_4_]^−^ counteranion omitted for clarity).

Schneider and Holthausen recently disclosed a multistep *in crystallo* reaction sequence in the context of the Wolff rearrangement.^53^ *In crystallo* photolysis of Pt diazo complex **18** at 80 K afforded carbene **19**. Subsequent warming of the crystal to 120 K induced rearrangement to the ketenyl complex **20** (Figure 4). While the ketenyl species evolved further to formyl complex **21** during solution phase photolysis, this transformation was not observed *in crystallo*, presumably due to loss of crystallinity induced by evolution of three gaseous small molecules (i.e., **18** → **21** + N_2_ + CO + C_2_H_4_) within a nonporous lattice. Together with the aforementioned multistep sequences, these data highlight that *in crystallo* cascades can be accomplished either by sequential photoreactions with different excitation wavelengths or by thermal annealing of photogenerated reactive crystals.

**Figure 4:**
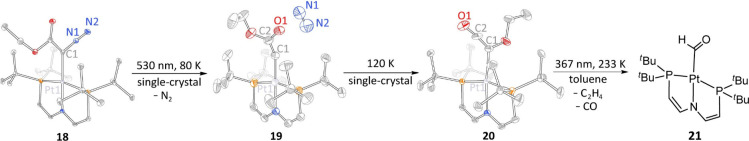
Multistep *in crystallo* reaction sequence defines the intermediates involved in the Wolff rearrangement.

Significant challenges remain to reliably and predictably characterizing multistep *in crystallo* reaction sequences. First, most of the photolabile ligands utilized for *in crystallo* studies operate via the extrusion of a gaseous small molecule, e.g., N_2_, CO, and CO_2_. During subsequent thermal annealing, which is often needed to promote *in crystallo* reactions of photogenerated reactive compounds, intracrystalline pressure and gas volatilization can lead to loss of crystallinity before sufficient chemical conversion can be achieved.^54^ Second, obtaining sufficiently pure crystalline samples is a challenge. Often, *in crystallo* photoreactions do not proceed to completion and thus the primary photostructures are necessarily refined as two-component compositional disorder models of photoprecursor and photoproduct.^42,52^ In a multistep reaction sequence, if the reaction of the photogenerated compound does not proceed to completion, the resulting crystalline sample is composed of three distinct species. Complex sample composition can prevent meaningful data analysis. Finally, to capture the dynamic processes evoked by “molecular movies”, the application of time-resolved methods is a frontier challenge. The crystallographic (and accompanying spectroscopic) experiments described in this Outlook are steady-state measurements in which photoproducts are first generated within the single-crystal environment and then characterized. While time-resolved diffraction methods have been developed to examine transiently generated species,^55^ thus far, these methods have not been adopted by the synthetic community.

### 3.2 New Synthetic Logic

The boundaries of synthetic tractability are inherently coupled to the availability (or lack thereof) of the appropriate reagents to promote a particular transformation. In the context of solution-phase synthesis, prolonged synthetic efforts have resulted in an array of designer reagents for atom- and group-transfer reactions. Similar efforts have not yet been directed toward photolabile ligands, which are critical to realizing the potential impact of *in crystallo* chemistry.

In the context of *in crystallo* nitrene photochemistry, organic azide ligands are a seemingly obvious choice for a photoprecursor, but highlight some oft-encountered challenges: 1) Organic azides are weak Lewis bases and thus weakly bind to many transition metal centers, 2) N_2_ loss can be thermally promoted and facile, rendering potential photoprecursors unstable toward spontaneous nitrene formation (and consumption), and 3) the evolution of N_2_ can prevent subsequent thermally activated reaction chemistry (*vide supra*). To broaden photochemical access to metal nitrenes and inspired by the burgeoning N–X photoactivation literature in organic chemistry, we introduced *N*-haloamides as nitrene photoprecursors.^56,57^ Unlike azides, which are L-type ligands (i.e., neutral donors), haloamides are X-type ligands (i.e., anionic donors) and thus coordinate strongly to many Lewis acid transition metal centers. Solution phase studies have demonstrated that photoactivation of Rh_2_[II,III] and Mn­(III) haloamide complexes **22** and **24** can provide entry to metal nitrenes **23** and **25**, which provides a new synthetic disconnection to these reactive intermediates (Figure 5).^56,57^ At present, this promise has not been successfully translated to *in crystallo* experiments, presumably because the photoeliminated halogen radical causes sample decomposition.

**Figure 5:**
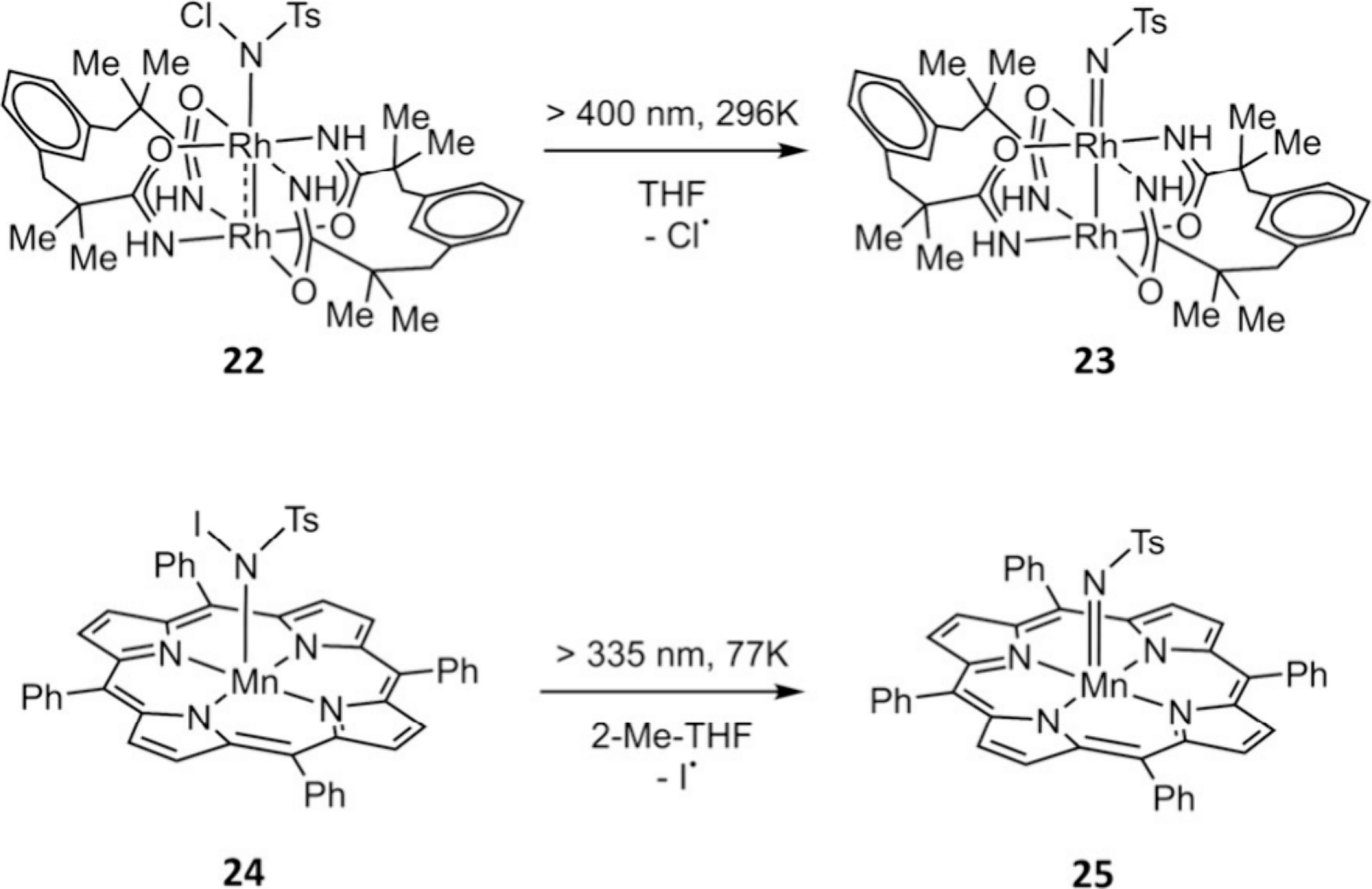
Design of *N*-haloamides as a new class of photolabile nitrene precursors highlights the potential to adapt well-developed organic photoreactions to *in crystallo* synthesis.

A second area of pressing need is the development of photoprecursors for *in crystallo* synthesis of metal oxo and oxyl complexes, which have enjoyed a central role in the development and application of molecular inorganic chemistry.^58^ At present, efficient photochemical processes to generate these species in the solid state are not available. Both Powers^59^ and Nocera^60^ have explored the photoactivation of Cu­(II) oxyanion complexes (bromate and chlorate, respectively) in an effort to access Cu–oxyl species. Thus, far, these studies have been plagued by competitive photoreduction (i.e., formation of Cu­(I) in preference for Cu–O species) and low photoconversion, which renders structure assignment challenging. Given the demonstrated successes in the context of metal nitrene chemistry, we view the development of oxo photoprecursors as the most significant roadblock to translating *in crystallo* photochemistry to the synthesis and characterization of highly reactive M–O fragments.

We view the extensive canon of well-defined organic photoreactions to be a source of inspiration for the development of novel photolabile ligands for *in crystallo* synthesis.

### 3.3 Demand for *In Crystallo* Spectroscopy

Widespread adoption of *in crystallo* synthetic chemistry as a paradigm to study reactive and exotic molecular targets requires the simultaneous deployment of *in crystallo* spectroscopy. Questions of sample composition, chemical structure, and electronic configuration can often not be addressed by crystallographic data alone. While many of the spectroscopic tools highlighted below are commonplace in solution-phase synthetic studies, the technical demands of deploying in single crystal reaction environments is often nontrivial.

#### 3.3.1 Establishing Composition

Characterization of sample composition during *in crystallo* photochemistry often requires real-time spectroscopy. For example, during the *in crystallo* conversion of Fe azide **26** to nitrene **27** described by Meyer and Albrecht (Figure 6a), the relative populations of starting material and products slowly and continuously changed over the course of photolysis.^61^ Because crystallography is an ensemble measurement (i.e., not spatially resolved within the single crystal), the data obtained is a superposition of **26** and **27**. Accurate determination of the relative populations of these two molecules in the lattice, especially at early reaction times, is challenging based on crystallography alone. Complementary Mössbauer spectroscopy enabled unambiguous refinement of a two-component reaction system (i.e., only **26** and **27** in the crystalline sample) (Figure 6b). In this case, challenges with bulk photolysis of solid-state samples (i.e., efficient penetration of light into a solid-state sample) mandated that the Mössbauer studies be carried out with a frozen solution of **26**. Solid-state NMR is another potentially valuable tool for probing compositional changes during photochemical reactions in the crystal lattice.^62,63^ However, achieving sufficient photoconversion through bulk photolysis can be challenging.

**Figure 6:**
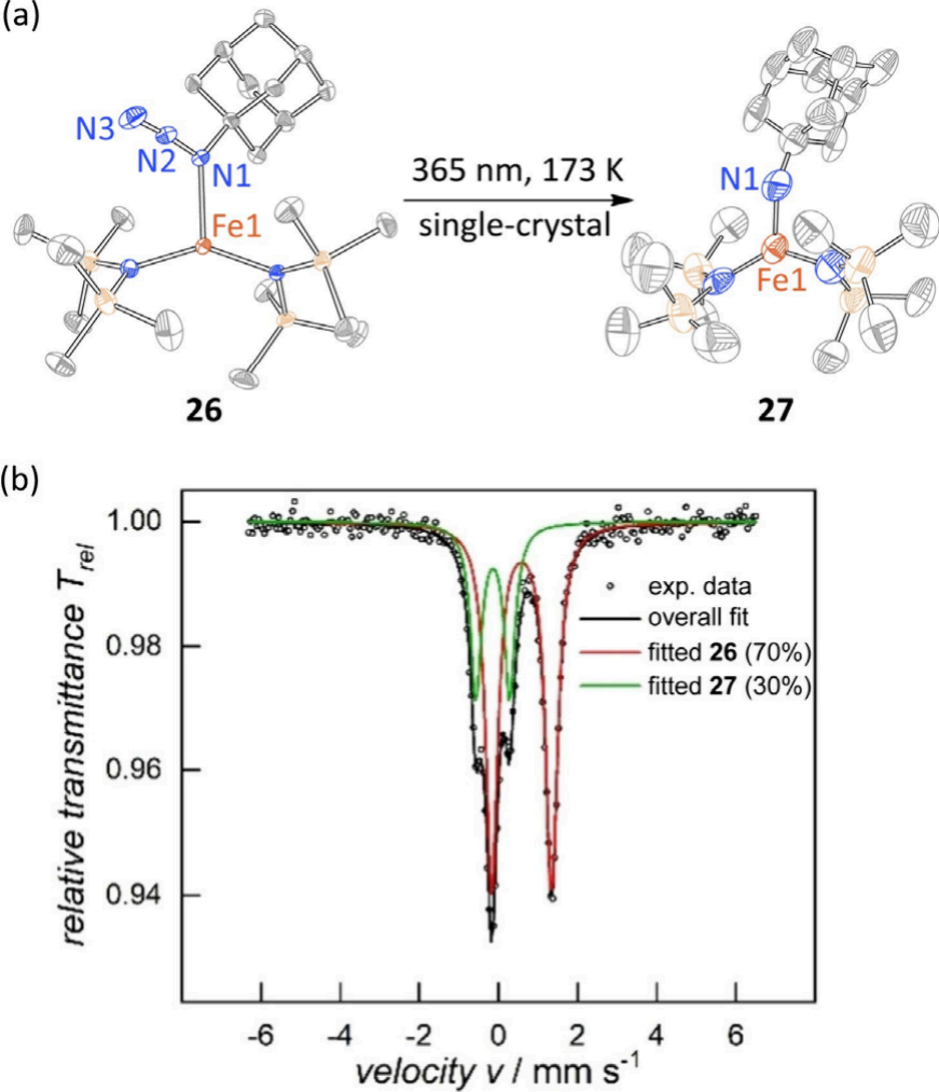
(a) *In crystallo* N_2_ photoelimination from **26** unveils Fe nitrene **27**. (b) The relative populations and electronic properties of **26** and **27** were elucidated by Mössbauer spectroscopy. Figure 6b is adapted with permission from ref 61. Copyright 2024 Wiley-VCH.

#### 3.3.2 Establishing Chemical Structure

Refinement of atomic positions for molecular fragments containing elements with similar atomic numbers (e.g., the N and the O atoms in nitrosyl ligands) can be challenging for crystallography. Similarly, the presence of protons of potential reactive ligands (e.g., metal–oxo vs hydroxide) is often implied, not directly refined. In these cases, single-crystal vibrational^64^ and electronic absorption^65^ spectroscopies can provide insight into M–L bonding modes and potential protonation state that is challenging from crystallography alone. For example, while the photoconversion of an *N*-bound nitrite complex to a metastable *O*-bound nitrito isomer is straightforward to assign by crystallography (i.e., change in connectivity of a triatomic unit),^66^ *in crystallo* nitrosyl-isonitrosyl linkage isomerism in *fac*-[RuNO­(NO_2_)_2_Py_2_OH] is more challenging. In this case, *in crystallo* linkage isomerism was corroborated by single-crystal IR and Raman spectroscopies.^67^ Analogous single-crystal UV–vis spectra enabled real-time monitoring of the *in crystallo* linkage isomerism of Ru–SO_2_ complex **28** (Figure 7).^65^ Beyond providing confirmation of the chemical structure, vibrational and optical spectra provide additional experimental benchmarks to compare with computational models.

**Figure 7:**
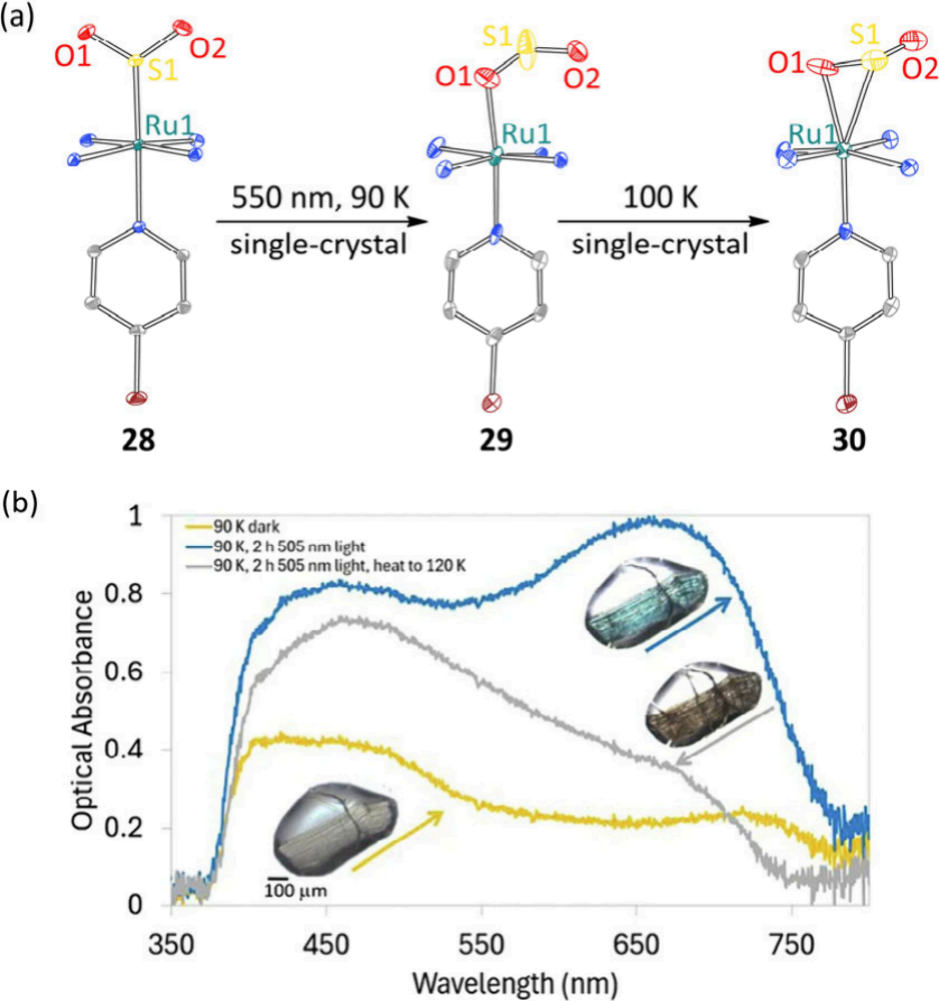
(a) *In crystallo* linkage isomerism of the SO_2_ ligand in Ru–SO_2_ complex **28**. (b) Single-crystal UV–vis spectra of **28**, isomer **29** obtained after photolysis, and isomer **30** obtained after heating. Figure 7b is reproduced from ref 65. Available under a CC BY 4.0 License (http://creativecommons.org/licenses/by/4.0/). Copyright 2025 The Authors.

In addition to steady-state spectroscopies, significant advances in understanding solid-state reaction mechanisms have come from transient absorption spectroscopy of nanocrystalline suspensions.^68,69^ While diffuse reflectance laser flash photolysis has been available for some time, the greater ease and reliability of acquiring transient spectra from nanocrystalline suspensions have made reactivity studies far more accessible.^70,71^ Extending this technique to transition metal photoreactions could provide a powerful spectroscopic tool for studying *in crystallo* transformations.

#### 3.3.3 Establishing Configuration

Application of *in crystallo* synthesis to increasingly exotic structures demands complementary spectroscopy to corroborate and characterize the proposed structures. For example, in the context of high-spin M–L complexes, spectroscopic data are critical to electronic structure predictions. In the context of metallopnictinidene chemistry, only small perturbations in the M–E distance are observed upon photoconversion of precursor molecules.^42^ While these observations are consistent with high-level calculations of triplet configurations, magnetic measurements, via either SQUID magnetometry^40,41,72^ or THz-EPR spectroscopy,^42^ were critical to establishing the proposed triplet configuration. Similar to the Mössbauer studies described above, while both magnetometry and EPR measurements can be carried out on photolyzed single crystals, often glassy solvent matrices are employed and provide a bridge between solution-phase and single-crystal habits.

## 4 Promise

X-ray crystallography continues to be the gold standard in small molecule structure determination. In many ways, the ability to acquire a crystal structure—to be able to prepare sufficient quantities of a target substance in sufficient purity to crystallize and unambiguously assign molecular structure—defines the limits of synthetic tractability. Historically, reactive species have not been amenable to crystallographic characterization simply because of the insufficient lifetime to be crystallized. In response, models of reactive structures are often studied in which synthetic derivatization attenuates the reaction of the species of interest, and structure–function relationships are constructed inferentially.

The advent of *in crystallo* photochemistry provides an opportunity to challenge long-held views of the boundaries of synthetic tractability. By combining crystalline molecular confinement with cryogenic photochemistry, *in crystallo* photochemistry has already enabled observation of fleeting intermediates in catalytic reactions, exotic electronic structures that give rise to new reaction mechanisms, and provided a venue for distorted molecular geometries and the impact of those distortions on reactivity.

In our opinion, these early successes point to a bright future for *in crystallo* photosynthesis.

The ability to isolate and characterize short-lived species should stimulate new efforts to conceive of molecular targets and to imagine new photochemical routes for their synthesis.

The potential to effect multistep chemical reactions within a crystal lattice should inspire the recording of *bona fide* molecular movies that chart atomic positions throughout a chemical reaction. The opportunity to leverage lattice confinement to access nonground state geometries should stimulate efforts to devise new platforms to generate distorted species in substrate-accessible lattices and inspire efforts to systematically explore chemistry inaccessible in solution. In our view, the successes demonstrated thus far illuminate a world of new opportunities (and associated challenges) that promise to redefine the boundaries of synthetic tractability.
